# Acute Management of Neurological Events in Mitochondrial Encephalomyopathy, Lactic Acidosis, and Stroke-Like Episodes (MELAS) Syndrome: A Case Report

**DOI:** 10.7759/cureus.83959

**Published:** 2025-05-12

**Authors:** Zaza Aladashvili, Thalia B Rodriguez, Guillermo Izquierdo-Pretel

**Affiliations:** 1 Internal Medicine/Critical Care, Jackson Memorial Hospital, Miami, USA; 2 Faculty of Medicine, Tbilisi State Medical University, Tbilisi, GEO; 3 Medicine, Ross University School of Medicine, Miramar, USA; 4 Internal Medicine, Jackson Memorial Hospital, Miami, USA; 5 Hospital Medicine, Jackson Memorial Hospital, Miami, USA; 6 Internal Medicine, Florida International University, Herbert Wertheim College of Medicine, Miami, USA

**Keywords:** arginine therapy, case report, lactate, melas syndrome, metabolic dysfunction, mitochondrial disease, multidisciplinary care, seizures, stroke-like episodes

## Abstract

Mitochondrial encephalomyopathy, lactic acidosis, and stroke-like episodes (MELAS) is a rare mitochondrial disorder characterized by recurrent stroke-like episodes, seizures, and progressive neurological decline. We presented the case of an 18-year-old female, diagnosed with MELAS syndrome at age 11, who presented with acute vision loss and seizures. Neuroimaging revealed acute infarcts in the occipital and parietal lobes, consistent with MELAS syndrome-related strokes. Elevated lactate levels confirmed metabolic dysfunction. Management included arginine supplementation, seizure medication optimization, and a high-fat, low-carbohydrate diet. The patient's vision improved, seizures subsided, and lactate levels normalized. This case highlights the importance of early recognition and a multidisciplinary approach in optimizing the metabolic and neurological management of MELAS syndrome.

## Introduction

Mitochondrial encephalomyopathy, lactic acidosis, and stroke-like episodes (MELAS) is a rare, maternally inherited mitochondrial disorder predominantly affecting the muscles and nervous system [1]. The disorder is primarily caused by point mutations in mitochondrial DNA (mtDNA), particularly the m.3243A>G mutation or m.3271T>C in the MT-TL1 gene, which disrupts oxidative phosphorylation and ATP production [2]. The m.3243A>G mutation accounts for about 80% of cases, while m.3271T>C accounts for 10% [3,4]. People with m.3271T>C have an earlier onset of clinical manifestations, which is often seen in infancy. Compared to the m3243A>G group, they have more severe neurologic symptoms [3]. MELAS syndrome has an estimated prevalence of 0.18 per 100,000 and an incidence of 1 in 4,000 [1,2,5]. It affects both genders equally, and the age of onset is typically before age 20 [6]. It presents with a wide range of symptoms, including seizures, myopathy, recurrent migraines, vomiting, short stature, and hearing loss [1]. Additional manifestations may include diabetes, cardiac disease, and psychiatric symptoms [1,7].

Pediatric MELAS syndrome typically presents with failure to thrive, developmental delay, seizures, and early stroke-like episodes, whereas adult-onset cases tend to feature progressive neurological decline, muscle weakness, diabetes, and psychiatric symptoms [1,2]. The variable presentation of MELAS syndrome can mimic other neurological disorders, such as multiple sclerosis, peripheral neuropathy, or a vascular stroke [8], complicating its management. Lactate monitoring plays a huge role in monitoring this disease. It is the initial indicator for stroke-like episodes and sometimes can be misdiagnosed with tissue hypoxic-ischemic injury [1,9]. But the difference is that with MELAS syndrome, there is increased lactate and normal O2 saturation [1]. Whereas hypoxic-ischemic injury manifests with increased lactate in the presence of decreased O2 levels [1]. There is no actual treatment that can stop the progression of the disease. The goal is to manage patients symptomatically, such as through seizure management and mitochondrial supplements: CoQ10, arginine, Citrulline [1,6]. This case report aims to contribute to the limited literature on the acute management, metabolic therapy, and multidisciplinary approach to MELAS syndrome.

## Case presentation

An 18-year-old female with a history of MELAS was diagnosed at age 11. Genetic testing confirmed the diagnosis, identifying the mtDNA m.3243A>G pathogenic variant with 53% heteroplasmy. The patient's medical history included recurrent strokes, axonal peripheral neuropathy, mild neurosensory hearing loss, sensory ataxia, and a history of seizures. Home medications were oxcarbazepine, brivaracetam, and clobazam for controlling the seizure episodes. Her intellectual development was impacted early, coinciding with the onset of her MELAS syndrome symptoms. Currently, she is in Grade 12, receiving homeschooling and special education support. The patient had a family history of seizures, with her 15-year-old brother also likely affected by MELAS syndrome; however, no genetic testing had been performed to confirm the diagnosis. She had two half-brothers on her mother’s side, both of whom were healthy. Genetic testing of the parents revealed no mitochondrial disorders, and their medical histories showed no chronic illnesses.

The patient experienced blurry vision in the morning, which rapidly progressed to complete vision loss, prompting her to seek medical attention. While at the ophthalmology clinic, she developed seizures, leading to her hospital admission. She presented to the emergency department with seizure-like activity and worsening vision loss. She had four episodes of rhythmic jerking movements of the head and eyes, each lasting 30 seconds. Despite experiencing seizure-like movements, the patient remained alert. The rapid loss of vision raised concerns about a potential acute neurological event, further prompting an ophthalmologic evaluation.

**Figure 1:**
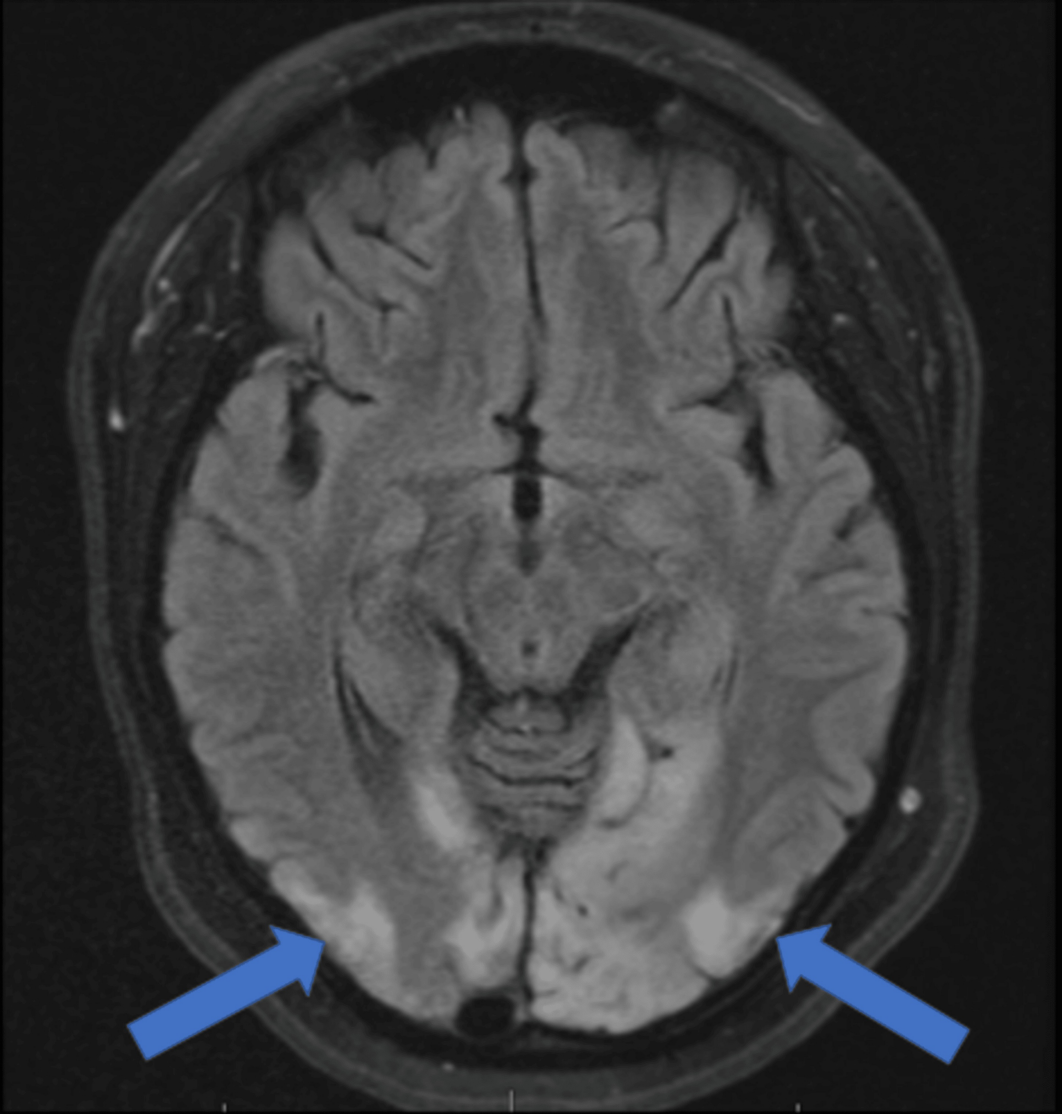
Axial fluid-attenuated inversion recovery (FLAIR) brain MRI. The image demonstrates cortical and subcortical hyperintensities (indicated by arrows) predominantly in the bilateral occipital lobes, more marked on the left, consistent with a stroke-like episode. These findings are characteristics of mitochondrial encephalopathy and do not conform to vascular territory.

A CT scan performed upon initial evaluation revealed hypodensities in the bilateral occipital and left parietal lobes, suggestive of age-indeterminate infarcts, as well as right occipital encephalomalacia. This raised suspicion of MELAS-related strokes. Further diagnostic imaging was conducted with an MRI, which demonstrated acute ischemic changes, including diffusion restriction and edema in the left occipital and parietal lobes, consistent with acute ischemia due to MELAS-associated strokes. Chronic ischemic changes were also noted in the right occipital lobe (Figure 1).

Additionally, laboratory tests revealed an elevated lactic acid level on the day of admission, which decreased to a normal range on day 2, indicating some improvement in the patient's metabolic status.

Over the following two days, the patient's condition improved significantly. By the third day of admission, her vision had begun to recover, and she was able to differentiate between numbers and colors, which indicated a positive response to treatment. No further seizures were observed during her hospital stay, suggesting stabilization of her condition. The hospitalist and neurology teams closely monitored the patient throughout her hospitalization. The overall clinical course was favorable, with the patient showing signs of improvement in both her neurological and vision status.

**Table 1:** Daily progression of lactate levels. This table shows the lactate level during admission and its daily progression after the management of the patient's acute symptoms and medication administration.

Day 1	Day 2	Day 3	Reference range
3.5 mEq/L	2.6 mEq/L	2.3 mEq/L	0.5-2.2 mEq/L

The patient was discharged with a comprehensive management plan that emphasized outpatient follow-up. Discharge recommendations included adherence to a high-fat, low-carbohydrate diet, continued hydration, and monitoring for any recurrence of seizures or vision changes. For seizure management, clobazam, brivaracetam, and lacosamide were prescribed, and a neurology follow-up was scheduled. Additionally, the patient was advised to follow up with the genetics department for long-term management and further recommendations. An outpatient genetics consultation was scheduled to assess her mitochondrial function and review ongoing treatment options, including potential modifications to her supplementation regimen. The importance of regular monitoring of her lactic acid levels and plasma amino acid levels was also emphasized.

## Discussion

The pathophysiology of MELAS syndrome involves mitochondrial dysfunction, leading to impaired oxidative phosphorylation, energy production deficits, and increased oxidative stress [6]. This leads to increased anaerobic glycolysis and elevated lactate levels, contributing to metabolic acidosis and tissue damage [1]​. In smooth muscle, mitochondrial proliferation can stimulate angiopathy and contribute to observed complications such as stroke-like symptoms [6,10]. Brain imaging in MELAS syndrome typically shows initial lesions in the occipital, parietal, and temporal lobes, which mimic ischemic strokes but do not conform to vascular territories [1,4]. Occipital lobe lesions, as seen in this patient, leave the eye intact but result in cortical blindness rather than ocular vision loss.

Lactate monitoring is vital for managing MELAS syndrome, as it helps assess disease progression and evaluate treatment effectiveness. It can be a diagnostic factor for acute stroke-like episodes [1]. Elevated lactate levels signal mitochondrial dysfunction, allowing clinicians to adjust interventions accordingly [1]. Metabolic support, such as arginine, CoQ10 supplementation, and dietary changes (e.g., ketogenic diet), improves mitochondrial function and reduces lactate levels. Arginine is a precursor of nitric oxide (NO); its function is to vasodilate arteries. Increased arginine can elevate levels of NO, which enhances blood circulation in the brain. Therefore, more oxygen can be delivered to that area, decreasing the chance of developing stroke-like episodes [1]. Additionally, CoQ10 boosts energy production in mitochondria and decelerates the progression of MELAS [1], which is essential for symptom management and preventing further neurological decline [6]. Numerous studies suggest that elevated lactate can contribute to cardiogenic and hypovolemic shock, altered mental status, and tachypnea, highlighting lactate control as a key mechanism in managing MELAS syndrome dynamics [9].

On the other hand, seizures are a common and serious feature of MELAS syndrome, often occurring as focal seizures, status epilepticus, or during stroke-like episodes. Prompt seizure control reduces metabolic stress and prevents further brain injury. Certain antiseizure drugs, such as valproic acid, phenytoin, carbamazepine, and phenobarbital, are avoided due to their mitochondrial toxicity. Safer options, like levetiracetam, brivaracetam, lamotrigine, and clobazam, are preferred. Our patient was discharged on clobazam, brivaracetam, and lacosamide for their effectiveness and safety in mitochondrial disease, providing seizure control without worsening metabolic dysfunction.

Effective management requires a multidisciplinary approach involving neurology, ophthalmology, genetics, and nutrition. Neurologists manage neurological symptoms, ophthalmologists address vision problems, geneticists guide long-term treatment, and nutritionists support mitochondrial health. This collaboration improves outcomes and ensures comprehensive care. Our case highlights the success of this multidisciplinary approach in managing the complex presentation of this patient.

Assessing treatment efficacy is challenging due to the disorder's progressive nature and the possibility of spontaneous remission. MELAS syndrome is associated with a high mortality rate, particularly in patients with symptom onset before six months of age. In one long-term study, the average age of death was 34.5 years, with 22% of deaths occurring before age 18, and another study found that 20.8% of patients died within a median of 7.3 years from diagnosis [11]. While supportive therapies improve quality of life, no current interventions have been shown to reduce mortality in MELAS. The disease exhibits heteroplasmy, with clinical manifestations varying based on the percentage of affected mitochondrial cells, complicating management [2,8]. Which often relies on anecdotal evidence and expert consensus rather than controlled studies.

Further research is essential to standardize care and develop evidence-based guidelines for MELAS syndrome management. As current management relies on expert opinion and case reports, more controlled studies are needed to better understand the disease's progression and define the most effective therapeutic strategies.

## Conclusions

Early recognition and timely management are crucial for optimizing outcomes for patients with MELAS syndrome. Given its broad spectrum of clinical manifestations, a symptom-specific, multidisciplinary approach is vital. Coordinated care among neurology, genetics, ophthalmology, and nutrition teams enhances treatment efficacy and mitigates complications. In addition to pharmacologic therapy, nutritional support plays a vital role in maintaining metabolic stability and reducing the risk of recurrence.

The rarity of MELAS syndrome and the absence of standardized treatment guidelines present significant challenges for clinicians. Ongoing research and the accumulation of well-documented case reports are essential to develop evidence-based protocols and improve long-term patient care.

## References

[1] Pia S, Lui F (January 25, 2024). Melas Syndrome. https://www.ncbi.nlm.nih.gov/books/NBK532959/.

[2] Barros CD, Coutinho A, Tengan CH (2024). Arginine supplementation in MELAS syndrome: what do we know about the mechanisms?. Int J Mol Sci.

[3] Seed LM, Dean A, Krishnakumar D, Phyu P, Horvath R, Harijan PD (2022). Molecular and neurological features of MELAS syndrome in paediatric patients: A case series and review of the literature. Mol Genet Genomic Med.

[4] Alsultan M, Alshaar D, Alkhouli B, Hassan Q (2022). MELAS syndrome with rare manifestations misdiagnosed as vasculitis in the absence of lactic acidosis: a case report. Ann Med Surg (Lond).

[5] Xu S, Jiang J, Chang L, Zhang B, Zhu X, Niu F (2024). Multisystem clinicopathologic and genetic analysis of MELAS. Orphanet J Rare Dis.

[6] Yatsuga S, Povalko N, Nishioka J (2012). MELAS: a nationwide prospective cohort study of 96 patients in Japan. Biochim Biophys Acta.

[7] El-Hattab AW, Almannai M, Scaglia F (2017). Arginine and citrulline for the treatment of MELAS syndrome. J Inborn Errors Metab Screen.

[8] Koga SJ, Hodges M, Markin C, Gorman P (1995). MELAS syndrome. West J Med.

[9] Foucher CD, Tubben RE (2023). Lactic Acidosis. https://www.ncbi.nlm.nih.gov/books/NBK470202/.

[10] Lorenzoni PJ, Werneck LC, Kay CS, Silvado CE, Scola RH (2015). When should MELAS (mitochondrial myopathy, encephalopathy, lactic acidosis, and stroke-like episodes) be the diagnosis?. Arq Neuropsiquiatr.

[11] (2025). MELAS and other mitochondrial disorders: a primer for physiatrists. https://www.upmcphysicianresources.com/news/melas-and-other-mitochondrial-disorders-a-primer-for-physiatrists.

